# GLP-1 Receptor Agonists as Molecular Relievers of Lipotoxic Stress: From Pancreatic Beta-Cell Cholesterol Efflux to Systemic and Tissue-Specific Metabolic Protection

**DOI:** 10.3390/ijms27188378

**Published:** 2026-09-20

**Authors:** Wenyi Jiang, Kensaku Fukunaga, Toshihiro Kobayashi, Takanobu Saheki, Takafumi Yoshimura, Haotian Zhang, Rathana Ly, Hitomi Imachi, Koji Murao

**Affiliations:** Department of Endocrinology and Metabolism, Faculty of Medicine, Kagawa University, 1750-1, Ikenobe, Miki-cho, Kita-gun 761-0793, Kagawa, Japan

**Keywords:** GLP-1 receptor agonist, lipotoxicity, pancreatic beta cell, ABCA1, cholesterol efflux, CaMKK/CaMKIV, PREB, endoplasmic reticulum stress, autophagy, metabolic dysfunction-associated steatotic liver disease

## Abstract

GLP-1RAs improve glycemia and body weight, but their effects extend beyond insulin secretion and appetite suppression. Experimental evidence indicates that GLP-1 receptor signaling can relieve lipotoxic stress by reducing lipid influx, restoring lipid trafficking, promoting cholesterol efflux, improving mitochondrial and endoplasmic reticulum homeostasis, and suppressing inflammatory and apoptotic signaling. In pancreatic beta cells, saturated fatty acids, oxidized low-density lipoprotein and excess free cholesterol disrupt membrane microdomains, insulin-granule trafficking, calcium signaling, autophagic flux and beta-cell identity. Preclinical studies with individual GLP-1RAs, principally exendin-4 and liraglutide, implicate cAMP/PKA, PI3K/Akt, ERK1/2, AMPK, Nrf2 and autophagy-related pathways in these protective responses. A relevant mechanism is induction of ATP-binding cassette transporter A1 (ABCA1): exendin-4 stimulates ABCA1 transcription through the CaMKK/CaMKIV/PREB axis, linking incretin signaling to cholesterol export and preservation of glucose-stimulated insulin secretion. Recent work indicates spatially organized GLP-1R signaling at endoplasmic reticulum–mitochondria contact sites. Preclinical genetic evidence in mouse metabolic dysfunction-associated steatohepatitis (MASH) models indicates that pericentral liver sinusoidal endothelial GLP-1 receptors contribute to weight-loss-independent semaglutide-mediated improvements in steatosis, fibrosis and immune remodeling; whether an analogous causal mechanism operates in human MASH remains unknown. This review integrates systemic nutrient unloading, beta-cell cholesterol homeostasis and intrahepatic endothelial signaling as complementary mechanisms of metabolic protection.

## 1. Introduction

Type 2 diabetes and obesity develop in an environment of chronic nutrient excess in which adipose tissue buffering capacity becomes insufficient, circulating non-esterified fatty acids and atherogenic lipoproteins increase, and ectopic lipids accumulate in the pancreatic islets, liver, skeletal muscle, heart and kidney. The resulting cellular injury is commonly described as lipotoxicity. In pancreatic beta cells, lipotoxicity is not a simple consequence of total lipid content. It reflects the chemical species, duration of exposure and intracellular localization of fatty acids, ceramides, diacylglycerols and cholesterol, as well as the capacity of the cell to oxidize, esterify, store, traffic and export these lipids [1,2,3,4].

GLP-1 receptor agonists (GLP-1RAs) have transformed the treatment of type 2 diabetes and obesity. Their established actions include glucose-dependent enhancement of insulin secretion, suppression of inappropriate glucagon secretion, slowing of gastric emptying and reduction in energy intake [5,6]. These effects reduce the systemic nutrient load delivered to vulnerable tissues. Nevertheless, experimental studies indicate that GLP-1 receptor signaling also activates cell-protective programs that can directly oppose lipid-induced dysfunction. These include attenuation of endoplasmic reticulum (ER) stress, oxidative stress and apoptosis; restoration of autophagy; preservation of mitochondrial function; and reprogramming of lipid uptake, synthesis and efflux.

The authors’ previous ABCA1-focused work provides a mechanistic foundation for this broader concept. It identifies pancreatic ATP-binding cassette transporter A1 (ABCA1) as a gatekeeper of beta-cell cholesterol homeostasis and shows that exendin-4 increases ABCA1 transcription through Ca2+/calmodulin-dependent protein kinase kinase (CaMKK), CaM-dependent protein kinase IV (CaMKIV) and prolactin regulatory element-binding protein (PREB). This pathway connects GLP-1 receptor activation to cholesterol efflux, membrane integrity and insulin-granule exocytosis. The present review expands that framework and asks a broader question: can GLP-1RAs relieve lipotoxicity by coordinating lipid unloading and cellular stress adaptation across the beta cell and other metabolic organs?

## 2. Scope and Literature Search Strategy

This focused narrative review was designed to integrate experimental and translational evidence linking GLP-1 receptor signaling to cellular lipid handling and lipotoxic stress, with particular attention to pancreatic beta-cell cholesterol homeostasis and the authors’ previous work on the CaMKK/CaMKIV/PREB–ABCA1 pathway. PubMed/MEDLINE was searched from database inception through September 2026 using combinations of terms related to GLP-1 signaling and pharmacology (GLP-1, GLP-1 receptor agonist, endogenous GLP-1, exendin-4, liraglutide, semaglutide, receptor trafficking, biased agonism), nutrient-induced GLP-1 secretion (intestinal L cell, dietary protein, amino acid, unsaturated fatty acid, dietary fiber, short-chain fatty acid, D-allulose, rare sugar), lipotoxicity and cellular stress (lipotoxicity, glucolipotoxicity, ER stress, oxidative stress, mitochondrial dysfunction, mitochondrial dynamics, autophagy, ferroptosis, apoptosis), tissue-specific metabolic injury (pancreatic beta cell, hepatocyte, adipocyte, skeletal muscle, cardiovascular tissue, kidney, renal lipotoxicity, diabetic kidney disease, podocyte, proximal tubular cell, glomerular endothelial cell), and lipid-handling or signaling pathways (cholesterol, ABCA1, ABCG1, CaMKK, CaMKIV, PREB, ER–mitochondria contact sites, VAPB, SPHKAP, MICOS).

Priority was given to original mechanistic studies that directly interrogated the pathways discussed, particularly studies incorporating genetic or pharmacological perturbation, primary human cells or islets, tissue-selective models, or clinically relevant GLP-1 receptor agonists. Human mechanistic studies and randomized clinical trials were used to place experimental findings in translational context. When multiple publications addressed the same mechanism, preference was given to studies providing stronger causal evidence, more physiologically relevant models, or independent replication, while seminal earlier studies were retained when they established the original mechanistic concept. Reference lists of key primary studies and relevant recent reviews were manually screened for additional publications. The initial literature search was conducted in July 2026 and was updated in September 2026 to incorporate newly identified studies and evidence relevant to renal lipotoxicity and nutrient-induced endogenous GLP-1 secretion. The review was restricted to English-language publications because detailed critical appraisal required access to the full methodological and mechanistic descriptions. Because this article was designed as a focused narrative review, no formal PRISMA-based screening process or quantitative evidence synthesis was undertaken.

Throughout this review, the individual ligand or agonist used in each mechanistic study is specified wherever possible, and findings obtained with a single compound are not assumed to represent a class-wide GLP-1RA effect. Exendin-4 is retained as the term for studies that experimentally used this peptide; exenatide, the synthetic pharmaceutical form of exendin-4, is an FDA-approved GLP-1RA. Liraglutide and semaglutide are also FDA-approved GLP-1RAs, whereas native GLP-1 and the experimental biased agonists discussed in mechanistic studies are not FDA-approved therapeutic products. The regulatory context of the principal ligands discussed in this review is summarized in Supplementary Table S1.

### Use of Generative AI in Manuscript Preparation

**Figure 1:**
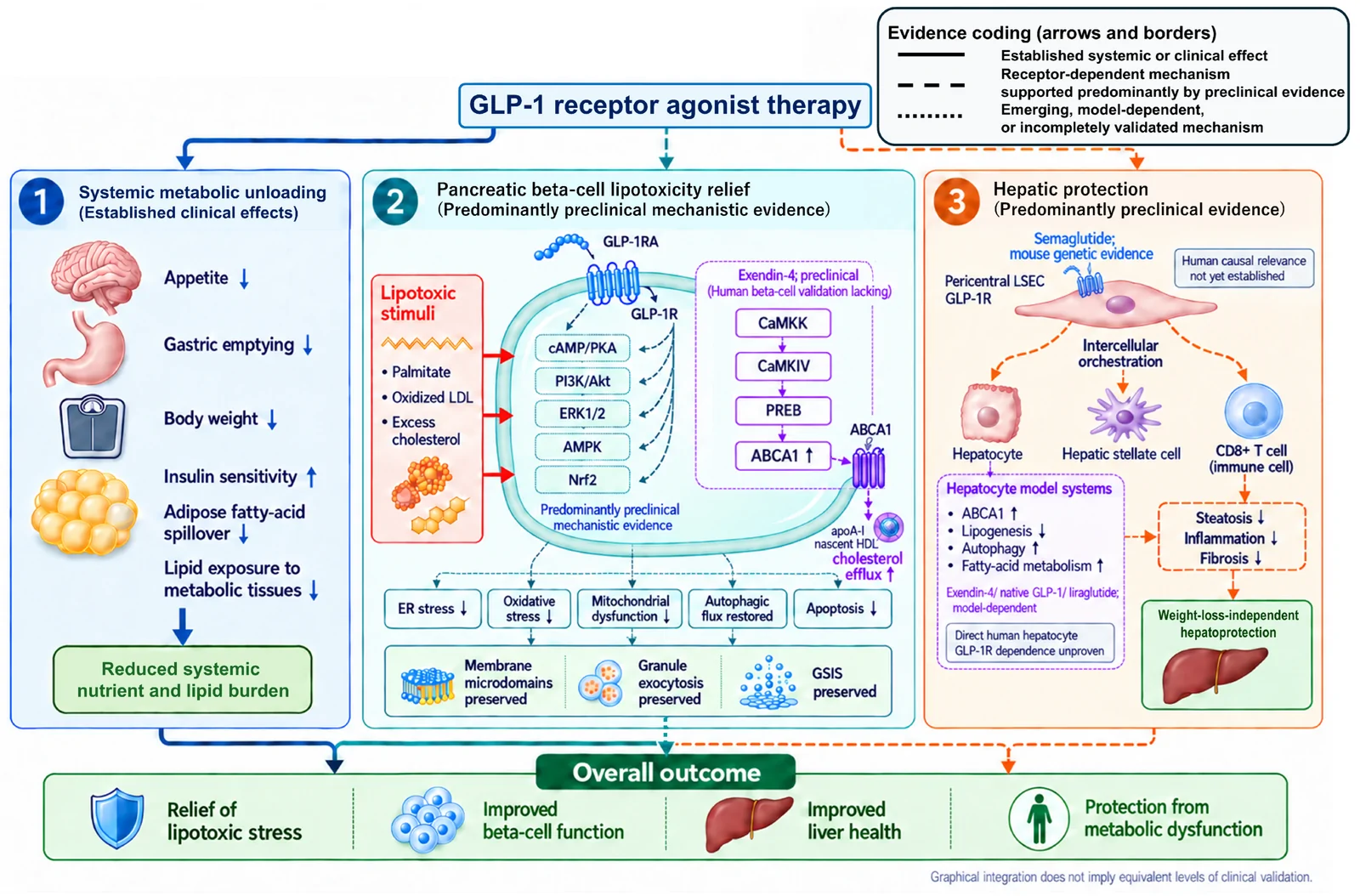
Evidence-calibrated integrated model of GLP-1 receptor agonist-associated relief of lipotoxic stress. GLP-1 receptor agonists reduce nutrient intake and body weight, decrease adipose fatty-acid spillover, and thereby lower lipid exposure to metabolic tissues. These systemic actions are clinically established and are shown with solid lines/borders. In pancreatic beta cells, GLP-1R-associated signaling through cAMP/PKA, PI3K/Akt, ERK1/2, AMPK, Nrf2, autophagy-related pathways, and spatially organized endosomal signaling at ER–mitochondria contact sites is supported predominantly by preclinical mechanistic evidence and is shown with dashed lines/borders. The exendin-4–CaMKK/CaMKIV–PREB–ABCA1 pathway is shown with dotted lines to indicate a compound-specific, incompletely validated preclinical mechanism; direct validation of GLP-1RA-induced ABCA1-dependent cholesterol efflux in human beta cells remains lacking. In mouse MASH models, semaglutide-responsive pericentral LSECs constitute a receptor-resolved intrahepatic signaling mechanism supported by genetic loss-of-function evidence and are shown with dashed lines/borders; whether a corresponding receptor distribution and causal LSEC signaling mechanism operates in human MASH remains to be established. In contrast, hepatocyte-model mechanisms involving ABCA1, SREBP-1c, autophagy, and lipid metabolism are shown with dotted lines because direct human hepatocyte GLP-1R dependence remains unproven. Solid lines/borders indicate established systemic or clinical effects; dashed lines/borders indicate receptor-dependent mechanisms supported predominantly by preclinical evidence; dotted lines/borders indicate emerging, model-dependent, or incompletely validated mechanisms. Graphical proximity does not imply equivalent levels of clinical validation. **Abbreviations:** ABCA1, ATP-binding cassette transporter A1; Akt, protein kinase B; AMPK, AMP-activated protein kinase; apoA-I, apolipoprotein A-I; CaMKK, Ca^2+^/calmodulin-dependent protein kinase kinase; CaMKIV, Ca^2+^/calmodulin-dependent protein kinase IV; cAMP, cyclic adenosine monophosphate; CD8, cluster of differentiation 8; ER, endoplasmic reticulum; ERK1/2, extracellular signal-regulated kinase 1/2; GLP-1, glucagon-like peptide-1; GLP-1R, glucagon-like peptide-1 receptor; GLP-1RA, glucagon-like peptide-1 receptor agonist; GSIS, glucose-stimulated insulin secretion; HDL, high-density lipoprotein; LDL, low-density lipoprotein; LSEC, liver sinusoidal endothelial cell; Nrf2, nuclear factor erythroid 2-related factor 2; PI3K, phosphoinositide 3-kinase; PKA, protein kinase A; PREB, prolactin regulatory element-binding protein.

For Figure 1, ChatGPT (OpenAI, GPT-5.6 Thinking) was used to assist with English-language editing and development of the initial visual layout. The authors subsequently reviewed, manually edited, and reconstructed the visual content using Adobe Illustrator (version 30.6) and Microsoft PowerPoint 2021 (version 2608) to ensure scientific accuracy, consistent terminology, and appropriate labeling. No GenAI tool was used to generate or analyze research data.

## 3. Lipotoxicity Is a Failure of Lipid Handling, Not Merely Lipid Excess

### 3.1. Fatty-Acid Species and Metabolic Context

Acute fatty-acid exposure can support beta-cell metabolism and amplify insulin secretion, whereas prolonged exposure, particularly to saturated fatty acids in the presence of hyperglycemia, promotes dysfunction and death. Palmitate induces endoplasmic reticulum (ER) stress, reactive oxygen species generation, mitochondrial depolarization, impaired autophagic degradation, inflammatory signaling and activation of pro-apoptotic BCL-2 family proteins. In contrast, monounsaturated fatty acids can be less toxic and may facilitate neutral-lipid storage. The concept of glucolipotoxicity therefore emphasizes that high glucose diverts fatty acids away from oxidation and toward harmful lipid intermediates while increasing the secretory burden imposed on the beta cell [1,2,3,4].

### 3.2. Cholesterol Lipotoxicity and Compartment-Specific Sterol Stress

Cholesterol is essential for plasma-membrane organization, secretory-granule biogenesis and exocytosis, but excess accessible cholesterol or abnormal intracellular distribution disrupts these same processes. Beta-cell-specific Abca1 deletion causes cholesterol accumulation, defective glucose-stimulated insulin secretion and glucose intolerance, and patients with ABCA1 mutations can show impaired insulin secretion [7,8]. Combined loss of ABCA1 and ATP-binding cassette transporter G1 (ABCG1) further worsens islet sterol accumulation, inflammation and secretory dysfunction [9]. Thus, beta-cell function depends on a balanced flux between low-density lipoprotein (LDL) receptor-dependent uptake, esterification, oxysterol-binding protein (OSBP)-mediated intracellular trafficking, proprotein convertase subtilisin/kexin type 9 (PCSK9)-dependent receptor regulation and ABCA1-dependent export. Recent work on beta-cell cholesterol redistribution and pancreatic PCSK9 further supports the importance of compartment-specific sterol handling [10,11].

This cholesterol-centered model widens the conventional definition of lipotoxicity. A beta cell can be exposed simultaneously to excess fatty acids, oxidized LDL and inflammatory cytokines. Oxidized LDL not only delivers modified lipid but also suppresses ABCA1 through mitogen-activated protein kinase kinase (MEK)/extracellular signal-regulated kinase (ERK)-dependent inhibition of liver X receptor (LXR), thereby disabling the adaptive efflux response [12]. Angiotensin II, tumor necrosis factor-α and N-methyl-D-aspartate signaling also suppress pancreatic ABCA1 [13,14,15]. Lipotoxic injury is therefore amplified when lipid influx is increased while protective export and trafficking pathways are impaired.

## 4. How GLP-1RAs Relieve Pancreatic Beta-Cell Lipotoxicity

### 4.1. Reduction in ER Stress and Apoptosis

Preclinical studies using individual GLP-1R agonists, principally exendin-4 and liraglutide, have demonstrated protection of beta cells against fatty-acid-induced apoptosis in multiple experimental systems. Exendin-4 reduces palmitate- or oleate-induced ER stress and apoptosis through induction of the ER chaperone binding immunoglobulin protein (BiP), the immediate-early transcription factor JunB and anti-apoptotic proteins, while limiting caspase activation [16]. Additional studies implicate phosphoinositide 3-kinase (PI3K)/Akt, extracellular signal-regulated kinase 1/2 (ERK1/2) and interference with free fatty acid receptor 1 (FFAR1/GPR40)–mitogen-activated protein kinase kinases 4/7 (MKK4/7)–c-Jun N-terminal kinase (JNK) stress signaling [17,18,19,20]. Exendin-4 also suppresses glucolipotoxic activation of sterol regulatory element-binding protein 1c (SREBP-1c) and CCAAT/enhancer-binding protein β (C/EBPβ), reducing ER stress and beta-cell apoptosis [21].

Liraglutide similarly attenuates ER stress and promotes beta-cell survival. Reported mechanisms include suppression of protein kinase R-like ER kinase (PERK)/eukaryotic initiation factor 2α (eIF2α) and inositol-requiring enzyme 1 (IRE1) signaling, induction of mesencephalic astrocyte-derived neurotrophic factor (MANF), and restoration of autophagy turnover [22,23]. Together, these compound-specific preclinical studies suggest that GLP-1 receptor (GLP-1R) activation may do more than acutely stimulate insulin secretion; under lipotoxic conditions, exendin-4 and liraglutide have been shown to reduce cellular stress and preserve the protein-folding and degradation systems required for sustained secretion.

ER stress may also change the way the GLP-1 receptor itself signals. In mouse islets, ER stress and diabetic conditions were reported to shift GLP-1R coupling from canonical Gs toward Gq, while activation of X-box binding protein 1 (XBP1) or activating transcription factor 6 (ATF6) selectively favored Gs utilization [24]. This observation introduces a reciprocal mechanism in which the lipotoxic/unfolded protein response (UPR) state can reshape receptor-proximal signaling, potentially altering the balance between cyclic adenosine monophosphate (cAMP)-dependent secretion, calcium mobilization and stress adaptation. The finding is mechanistically important but should not yet be generalized to human beta cells or to all long-acting GLP-1RAs.

### 4.2. Restoration of Autophagic Flux

Autophagy is an adaptive response that removes damaged organelles and lipid droplets, but prolonged saturated-fatty-acid exposure can block late-stage autophagic degradation. Liraglutide promotes macroautophagy in INS-1 cells and can activate AMP-activated protein kinase (AMPK)-dependent autophagic programs under metabolic stress [23,25,26]. Because palmitate causes accumulation of incompletely degraded autophagosomes, ER dilation and loss of insulin granules, restoration of autophagic flux represents a plausible mechanism contributing to the cytoprotective effects observed with liraglutide in these preclinical models.

### 4.3. Mitochondrial and Oxidative-Stress Protection

Beta cells have relatively limited antioxidant capacity and depend on mitochondrial coupling for glucose-stimulated insulin secretion. Lipotoxicity impairs respiratory efficiency, increases reactive oxygen species and destabilizes mitochondrial membrane potential. Exendin-4 activates nuclear factor erythroid 2-related factor 2 (Nrf2)-dependent antioxidant defense, reduces JNK and glycogen synthase kinase 3β (GSK3β) activity and preserves beta-cell viability under oxidative and palmitate stress [27,28]. Recent work further suggests that GLP-1 receptor agonism can preserve glucose-stimulated insulin secretion through a peroxisome proliferator-activated receptor δ (PPARδ)/uncoupling protein 2 (UCP2)-associated pathway: exendin-4 increased PPARδ, reduced UCP2, and improved mitochondrial membrane potential and mitochondrial DNA content, and these effects were lost when PPARδ was inhibited or deleted [29]. This positions mitochondrial coupling—not simply suppression of reactive oxygen species—as a mechanistic endpoint of anti-lipotoxic GLP-1R signaling. At the same time, current evidence does not establish a universal direction of UCP2 regulation across species or disease stages, and confirmation in human islets remains necessary. A 2026 synthesis similarly emphasizes mitochondrial morphology, redox control and organelle cross-talk as unresolved but potentially central dimensions of GLP-1 action in beta cells [30].

### 4.4. Spatially Organized GLP-1R Signaling Links Receptor Trafficking to Mitochondrial Remodeling

A major mechanistic advance is the recognition that GLP-1R signaling is not confined to the plasma membrane or to a spatially uniform cytosolic cAMP pool. In experiments performed predominantly with exendin-4, agonist-activated GLP-1R-positive endosomes were shown to dock at ER–mitochondria contact sites (ERMCSs) through interaction with the ER tether vesicle-associated membrane protein-associated protein B (VAPB) in beta-cell lines and primary mouse and human islets [31]. At these sites, GLP-1R engages sphingosine kinase type 1-interacting protein (SPHKAP), a protein kinase A regulatory subunit Iα (PKA-RIα)-selective A-kinase anchoring protein linked by human genetic studies to type 2 diabetes and adiposity. The resulting endosome–ER–mitochondria signaling assembly forms a localized cAMP/protein kinase A (PKA) hub and PKA-RIα biomolecular condensate that alters phosphorylation of the mitochondrial contact site and cristae organizing system (MICOS) complex, drives mitochondrial structural remodeling, and improves beta-cell insulin secretion and survival during ER stress [31].

The same study also compared native GLP-1 with the experimental exendin-4-based biased agonists exendin-F1 and exendin-D3, demonstrating ligand-dependent differences in receptor internalization and VAPB association [31]. Whether the complete VAPB–SPHKAP–MICOS signaling architecture and its cytoprotective consequences are quantitatively shared by clinically used GLP-1RAs remains unknown.

This spatial-signaling model provides a molecular bridge between receptor trafficking and organelle quality control. It also suggests that the anti-lipotoxic efficacy of a GLP-1RA may depend not only on receptor occupancy but also on the duration and subcellular location of signaling. GLP-1R agonists differ in G-protein versus beta-arrestin engagement, internalization and recycling, and 2025–2026 studies have refined the structural basis of biased agonism and shown that altered trafficking can prolong cAMP signaling [32,33]. However, whether G-protein-biased or trafficking-biased agonism specifically enhances protection against cholesterol, ceramide or saturated-fatty-acid stress in human beta cells has not yet been established. Biased agonism should therefore be regarded as an emerging mechanistic modifier of lipotoxicity relief rather than a proven anti-lipotoxic pathway.

### 4.5. ABCA1 Induction: A Candidate Cholesterol-Unloading Pathway

A mechanistically important question is whether GLP-1R signaling can relieve cholesterol stress by promoting ABCA1-dependent cholesterol handling. The biological relevance of ABCA1 to beta-cell function is well supported independently of GLP-1 signaling: beta-cell-specific Abca1 deficiency causes cholesterol accumulation and impaired insulin secretion in mice, whereas human carriers of ABCA1 loss-of-function variants have shown impaired beta-cell secretory function.

Within this framework, Li, Murao, Imachi and colleagues demonstrated that exendin-4 increased pancreatic ABCA1 transcription through CaMKK and CaMKIV in INS-1 cells and rat islets [34]. Miyai, Murao and colleagues subsequently identified PREB as a transcriptional mediator: exendin-4 increased PREB expression, PREB bound to and activated the ABCA1 promoter, and PREB silencing attenuated ABCA1 induction [35]. These studies define a specific exendin-4–CaMKK/CaMKIV–PREB–ABCA1 pathway.

Importantly, the broader concept that GLP-1 signaling can enhance ABCA1-associated cholesterol handling has received independent support through distinct upstream mechanisms. Yao and colleagues reported that GLP-1 reduced miR-27a expression and restored ABCA1 in cholesterol-loaded INS-1 cells, with concomitant reductions in lipid accumulation and beta-cell dysfunction [36]. Independently, Li and colleagues showed that exendin-4 activated AMPK, inhibited poly(ADP-ribose) polymerase 1 (PARP-1)-dependent repression of LXR, increased ABCA1 expression and cholesterol efflux, and attenuated cholesterol-induced beta-cell toxicity [37]. These studies suggest convergence of GLP-1 signaling on ABCA1 through multiple regulatory pathways rather than confirmation of a single universal upstream cascade.

The specific CaMKK/CaMKIV/PREB sequence, however, has been characterized primarily in studies from the authors’ group and, to our knowledge, has not yet been independently replicated in primary human islets. Likewise, direct demonstration that clinically used GLP-1RAs increase ABCA1 protein, apolipoprotein A-I (apoA-I)-dependent cholesterol efflux, or compartment-specific cholesterol removal in human beta cells remains lacking. Thus, the CaMKK/CaMKIV/PREB–ABCA1 pathway should currently be viewed as a mechanistically coherent but incompletely validated preclinical model, whereas the broader involvement of ABCA1 in beta-cell cholesterol homeostasis is supported by independent experimental and human genetic evidence.

## 5. Endogenous GLP-1 Signaling in Metabolic Stress: Intra-Islet and Nutrient-Responsive Pathways

GLP-1 relevant to beta-cell protection may arise not only from the intestine. Under lipotoxic stress, pancreatic alpha cells can increase prohormone convertase 1/3 expression and produce bioactive GLP-1. Experimental studies suggest that this intra-islet GLP-1 system acts as a local self-defense mechanism, limiting oxidative stress, inflammation and beta-cell death [38]. This concept places pharmacological GLP-1RA therapy within a physiological stress-response pathway: long-acting agonists may amplify an endogenous attempt by the islet to preserve beta-cell identity and function during nutrient overload. Beyond intra-islet GLP-1 production, endogenous GLP-1 secretion from intestinal L cells is also influenced by dietary signals, including protein and amino acids, unsaturated fatty acids, fermentable dietary fibers and their microbial metabolites, and the rare sugar D-allulose [39]. For example, oral D-allulose has been shown in preclinical models to induce GLP-1 release and activate vagal afferent GLP-1R signaling, contributing to reduced food intake and improved glucose tolerance [40]. Whether such food-mediated activation of endogenous GLP-1 signaling directly modifies cellular lipotoxicity remains unknown.

## 6. Hepatic Lipotoxicity: Systemic Unloading and Liver Sinusoidal Endothelial Cell-Centered Intrahepatic GLP-1 Receptor Signaling

### 6.1. Systemic Unloading and Weight-Loss-Independent Protection

GLP-1RAs reduce hepatic lipid burden through well-established systemic actions. Reduced energy intake and body weight, improved insulin sensitivity, lower glycemic exposure, decreased adipose-tissue fatty-acid spillover and improved lipoprotein metabolism all reduce substrate delivery to the liver. Clinical evidence indicates that GLP-1RA therapy can reduce liver fat and improve steatohepatitis activity [41,42]. More recently, in the phase 3 ESSENCE trial, semaglutide 2.4 mg increased both MASH resolution without worsening fibrosis (62.9% vs. 34.3%) and fibrosis improvement without worsening MASH (36.8% vs. 22.4%) at week 72 in adults with biopsy-confirmed metabolic dysfunction-associated steatohepatitis (MASH) and F2–F3 fibrosis [43]. Long-term liver-related clinical outcomes and efficacy in cirrhosis remain uncertain. These whole-body actions are genuine anti-lipotoxic mechanisms. However, recent evidence also indicates that hepatic protection cannot be explained solely by weight loss: semaglutide improved steatosis, fibrosis and immune remodeling in mouse MASH models resistant to GLP-1RA-induced weight loss, demonstrating a weight-loss-independent intrahepatic component [44].

### 6.2. Hepatocyte Model Responses, ABCA1, and Liver Sinusoidal Endothelial GLP-1 Receptor Signaling

Earlier cellular and animal studies reported that GLP-1 receptor agonism can modify hepatocyte lipid handling. In HepG2 cells and mouse hepatocyte models, exendin-4 reduced lipid accumulation and increased ABCA1 expression through the CaMKK/CaMKIV/PREB pathway [45]. In that experimental system, the GLP-1R antagonist exendin (9–39) attenuated exendin-4-induced ABCA1 expression, CaMKIV activation, and cholesterol reduction, providing pharmacological evidence consistent with GLP-1R-associated signaling. GLP-1 has also been reported to increase ABCA1 by downregulating miR-758 in cholesterol-loaded hepatocyte models [46]. Exendin-4 reversed hepatic steatosis in ob/ob mice [47], whereas liraglutide reduced hepatic lipid accumulation through AMPK/mechanistic target of rapamycin (mTOR)-associated induction of autophagy in high-fat-diet-fed mice and hepatocyte models [48].

These hepatocyte-model observations should, however, be distinguished from demonstration of a physiologically validated direct GLP-1R mechanism in human hepatocytes. Earlier studies reported GLP-1R expression and direct exendin-4 responses in human hepatocyte models [49], whereas more recent receptor-localization and functional studies have not consistently detected meaningful GLP-1R expression or agonist responses in human hepatocytes or hepatic stellate cells [50]. In particular, liraglutide did not reduce fatty-acid accumulation or elicit canonical CREB signaling in primary human hepatocytes in a recent study. Thus, pharmacological responses observed in HepG2 or other hepatocyte model systems remain mechanistically informative but cannot by themselves establish direct GLP-1R-dependent hepatocyte signaling in the human liver.

Recent cell-resolved and genetic studies provide an alternative framework for direct intrahepatic GLP-1 action. Gonzalez-Rellan and colleagues localized Glp1r expression in the mouse MASH liver predominantly to non-parenchymal populations, particularly pericentral liver sinusoidal endothelial cells (LSECs) and CD8+ T cells [44]. Semaglutide improved steatosis, fibrosis, and immune remodeling in mouse models resistant to GLP-1RA-induced weight loss, whereas endothelial Glp1r deletion or hepatic endothelial Glp1r knockdown substantially attenuated these hepatic benefits despite preserved systemic weight reduction. These findings support a GLP-1R-dependent, LSEC-centered intrahepatic signaling network in mouse MASH, in which receptor-bearing endothelial cells communicate with hepatocytes, stellate cells, and immune-cell populations. Importantly, whether an analogous receptor distribution and causal LSEC signaling mechanism operates in human MASH remains to be established.

Accordingly, hepatocyte ABCA1 induction and related lipid-handling responses are best regarded as experimentally observed, model-dependent mechanisms whose direct receptor dependence in human hepatocytes remains uncertain. In contrast, the LSEC-centered mechanism currently has stronger receptor-resolved causal support in mouse models, although its human translational relevance is not yet established.

## 7. Renal Lipotoxicity and Kidney Protection

Renal lipotoxicity is increasingly recognized as an important component of diabetic kidney disease. Excess fatty-acid uptake, enhanced de novo lipogenesis, impaired fatty-acid oxidation, and defective cholesterol efflux promote lipid accumulation in glomerular and tubular compartments, contributing to mitochondrial dysfunction, oxidative stress, inflammation, ferroptosis, and fibrosis [51,52].

Compound-specific preclinical studies suggest that GLP-1 receptor agonism may modify several of these processes. Liraglutide reduced renal tubular ectopic lipid deposition in diabetic rats and palmitate-treated tubular cells in association with AMPK activation, suppression of SREBP-1/FAS, and increased ATGL/HSL expression [53]. Exendin-4 has also been reported to reduce renal lipid accumulation and tubular ferroptotic injury through AMPK-linked fatty-acid metabolic pathways [52]. Particularly relevant to the cholesterol-efflux framework of this review, exendin-4 increased ABCA1 expression and cholesterol efflux in glomerular endothelial models and reduced renal cholesterol accumulation in diabetic apoE-deficient mice [54].

These observations remain predominantly preclinical and should not be interpreted as establishing a class-wide direct renal GLP-1R mechanism. Clinically, semaglutide reduces major kidney outcomes in patients with type 2 diabetes and chronic kidney disease [55], but the extent to which these benefits are mediated by direct modulation of renal lipid handling rather than systemic improvements in glycemia, body weight, hemodynamics, and inflammation remains unresolved.

## 8. Adipose Tissue, Skeletal Muscle and Cardiovascular Lipid Stress

Adipose tissue is the principal buffer for dietary lipid. GLP-1RAs reduce adipose mass primarily through lower energy intake and weight loss, thereby decreasing basal fatty-acid release and ectopic lipid delivery. Improved insulin action further suppresses inappropriate lipolysis. Direct effects on adipocytes are less consistently established and may vary by depot, species and receptor-detection method. In skeletal muscle, reductions in intramyocellular lipid and improved insulin sensitivity are likewise strongly influenced by weight loss, physical activity and lower circulating fatty acids. These systemic and tissue-level effects are summarized in broader mechanistic reviews of GLP-1-based therapy [5,6].

Cardiovascular protection may also involve relief of lipotoxic and inflammatory stress. GLP-1RAs reduce major cardiovascular events in high-risk populations [56], while experimental studies show improvements in endothelial function, oxidative stress, macrophage inflammation and myocardial substrate handling [5,6]. Nevertheless, organ-level outcomes integrate changes in weight, blood pressure, glycemia, renal function and systemic inflammation; they should not be attributed solely to direct lipid removal from cardiovascular cells.

## 9. An Integrated Model: Systemic, Receptor-Dependent, and Emerging Mechanisms of Lipotoxicity Relief

The mechanisms by which GLP-1 receptor agonist therapy may relieve lipotoxic stress can be organized into three conceptually distinct categories according to their biological level and strength of evidence. First, clinically established systemic effects reduce lipid exposure to vulnerable tissues. These include reduced energy intake and body weight, improved glycemic control and insulin sensitivity, and decreased adipose-tissue fatty-acid spillover [5,6]. These systemic actions lower nutrient and lipid delivery to the pancreatic islets, liver, kidney, skeletal muscle, and cardiovascular tissues and represent the most firmly established component of GLP-1RA-associated metabolic protection [5,6,41,42,43,56].

Second, tissue-specific receptor-dependent mechanisms have been demonstrated in defined experimental systems. In pancreatic beta cells, GLP-1R activation engages cAMP/PKA, PI3K/Akt, ERK1/2, AMPK, Nrf2, autophagy-related, and anti-apoptotic pathways that can reduce ER stress, oxidative injury, and mitochondrial dysfunction under metabolic stress [16,17,18,19,20,21,22,23,24,25,26,27,28,29,30]. Recent work has further linked endosomal GLP-1R trafficking at ER–mitochondria contact sites to VAPB–SPHKAP-organized local PKA signaling and MICOS-dependent mitochondrial remodeling in beta-cell lines and primary mouse and human islets [31]. In the liver, cell-resolved transcriptomic and genetic loss-of-function studies in mouse MASH models provide causal evidence that endothelial GLP-1Rs, particularly in pericentral LSECs, contribute to weight-loss-independent semaglutide-mediated improvements in steatosis, fibrosis, and immune remodeling [44]. These mechanisms are receptor-resolved and biologically compelling but remain predominantly preclinical, and their quantitative contribution to therapeutic responses in humans is not yet established [31,44].

Third, several direct cellular mechanisms remain promising but incompletely validated. These include exendin-4-induced CaMKK/CaMKIV/PREB-dependent ABCA1 expression in pancreatic beta-cell models [34,35], ABCA1 regulation through additional upstream pathways including AMPK/PARP-1/LXR signaling [37], hepatocyte-model responses involving ABCA1, lipogenesis, autophagy, and fatty-acid metabolism [45,46,47,48], and the possibility that biased agonism or altered receptor trafficking selectively enhances resistance to defined lipotoxic insults [32,33]. Independent experimental findings support the broader concept that GLP-1 signaling can converge on ABCA1 and cellular cholesterol handling through more than one upstream mechanism [34,35,37]. However, the specific CaMKK/CaMKIV/PREB sequence has not been established as a class-wide GLP-1RA mechanism or validated in primary human islets. Likewise, hepatocyte-model responses should not be interpreted as proof of direct GLP-1R-dependent signaling in human hepatocytes, particularly given recent studies questioning physiologically relevant GLP-1R expression and direct agonist responses in these cells [49,50].

This evidence-calibrated framework therefore separates reduction in systemic lipid exposure from receptor-dependent tissue signaling and from emerging model-dependent mechanisms. These categories are complementary rather than mutually exclusive: systemic nutrient unloading may reduce the magnitude of lipotoxic stress, whereas tissue-specific signaling may determine how individual cells adapt to the remaining lipid burden [5,6,31,34,35,37,44]. Table 1 summarizes the experimental and translational evidence for each mechanism, and Figure 1 depicts their integration while explicitly indicating the corresponding level of evidence.

## 10. Clinical Translation: Established Outcomes and Mechanistic Research Endpoints

Clinical translation of the anti-lipotoxic framework requires a clear distinction between established therapeutic outcomes and mechanism-specific research endpoints. GLP-1 receptor agonists produce clinically established reductions in body weight, glycemic exposure, and cardiovascular risk, and selected agents also improve organ-level outcomes relevant to lipid overload [5,6,43,55,56]. In MASH, for example, semaglutide improves histological disease activity and fibrosis-related endpoints [43], while randomized imaging studies demonstrate substantial reductions in hepatic fat measured by magnetic resonance imaging–proton density fat fraction (MRI-PDFF) [57]. These findings establish clinically relevant metabolic and tissue-level benefits but do not, by themselves, identify the cellular mechanisms responsible for those benefits. In particular, clinical outcome data do not establish that ABCA1-dependent cholesterol efflux, hepatocyte-autonomous signaling, or LSEC GLP-1R signaling mediates therapeutic efficacy in humans.

Mechanistic clinical studies should therefore incorporate endpoints that interrogate distinct levels of lipid handling. MRI-PDFF provides a quantitative tissue-level measure of hepatic lipid burden [57]. Circulating lipidomic profiling represents an emerging systemic approach: in adults with severe obesity, exenatide altered fasting and postprandial triglyceride, ceramide, and phospholipid species, with several changes persisting after adjustment for weight loss [58]. Cholesterol efflux capacity measured using apolipoprotein B (apoB)-depleted serum provides an established ex vivo measure of serum-mediated cholesterol acceptor function and captures information not reflected by HDL cholesterol concentration alone [59,60]. However, this assay reflects systemic cholesterol acceptor capacity and should not be interpreted as a direct measure of ABCA1 activity or cholesterol efflux in pancreatic beta cells.

Dynamic assessment of beta-cell function during standardized meal or glucose testing may provide a complementary physiological endpoint with which to relate changes in lipid exposure or lipid-handling biomarkers to insulin secretory function. Importantly, none of these measures is currently validated as a surrogate marker of GLP-1RA-mediated relief of beta-cell lipotoxicity. Prospective studies should determine whether changes in these mechanistic endpoints mediate improvements in beta-cell function or organ lipid burden independently of weight loss and glycemic improvement.

## 11. Limitations of Current Evidence

Several limitations constrain interpretation of the current evidence. First, much of the mechanistic literature is derived from insulinoma cell lines, rodent islets, or animal models, and many lipotoxicity protocols use fatty-acid concentrations or exposure conditions that do not directly reproduce human metabolic disease [1,2,3,4,16,17,18,19,20,21,22,23,24,25,26,27,28,29,30]. Experimental conditions also vary substantially with respect to fatty-acid species, albumin binding, glucose concentration, exposure duration, and the distinction between free cholesterol, esterified cholesterol, neutral lipid storage, and organelle-specific lipid distribution. These methodological differences limit direct comparison across studies.

Second, mechanistic evidence is frequently compound-specific. Many of the pathways discussed in pancreatic beta cells were demonstrated predominantly with exendin-4 or liraglutide rather than across the GLP-1RA class [16,17,18,19,20,21,22,23,24,25,26,27,28,29,30,31,32,33,34,35,37]. The CaMKK/CaMKIV/PREB–ABCA1 pathway, in particular, is supported mainly by exendin-4 studies and has not been independently validated as a class-wide mechanism in primary human islets [34,35,37]. Similarly, emerging data on biased agonism, receptor trafficking, and ER–mitochondria contact-site signaling are mechanistically compelling but remain incompletely characterized under clinically relevant agonist exposure [31,32,33].

Third, the cellular targets of GLP-1R signaling outside the pancreatic beta cell remain incompletely resolved. Direct GLP-1R expression and signaling in human hepatocytes remain controversial [49,50], whereas the LSEC-centered hepatic mechanism is supported by receptor-resolved genetic evidence in mouse MASH models but has not yet been causally established in the human liver [44]. Similar caution applies to emerging renal lipid-handling mechanisms, for which mechanistic evidence remains predominantly preclinical despite established clinical kidney benefits with selected GLP-1RAs [52,53,54,55].

Finally, organ-level clinical benefits cannot be assumed to identify the underlying cellular mechanism. Improvements in liver histology, kidney outcomes, cardiovascular risk, or glycemic control may reflect combinations of reduced energy intake, weight loss, altered circulating lipid flux, hemodynamic effects, and tissue-specific receptor signaling. Human mechanistic studies capable of separating these contributions remain limited.

## 12. Prioritized Future Research Agenda

A first priority is direct human validation of the proposed beta-cell cholesterol-handling mechanisms. Studies using primary human islets and stem-cell-derived beta cells should determine whether clinically relevant concentrations of individual GLP-1RAs increase plasma-membrane ABCA1 protein, apoA-I-dependent cholesterol efflux, and compartment-specific cholesterol redistribution. Genetic or pharmacological loss-of-function and rescue experiments will be necessary to establish whether ABCA1 is causally required for preservation of insulin secretion under cholesterol, oxidized-LDL, or saturated-fatty-acid stress [34,35,36,37]. Direct measurement of CaMKK/CaMKIV/PREB activation should further determine whether this specific exendin-4-associated pathway operates in human beta cells.

A second priority is to distinguish systemic metabolic unloading from tissue-specific receptor-dependent protection. Human and translational studies should incorporate weight-loss- and glycemia-matched comparators wherever feasible and should quantify circulating lipid flux together with tissue-specific outcomes. In the liver, studies should determine whether GLP-1Rs are reproducibly expressed in defined human LSEC subpopulations and whether endothelial activation produces measurable paracrine or circulating signals associated with histological improvement in MASH [44]. In the kidney, analogous studies should determine whether changes in renal lipid handling contribute independently to the established clinical renoprotective effects of GLP-1RA therapy.

A third priority is comparative analysis of receptor trafficking and spatial signaling across clinically used agonists. Semaglutide, liraglutide, exendin-4, and signaling-biased ligands should be compared at clinically relevant exposures using live-cell GLP-1R trafficking, compartment-resolved cAMP/PKA biosensors, ER–mitochondria contact-site imaging, and quantitative MICOS phosphoproteomics [31,32,33]. Such studies should determine whether differences in internalization, recycling, beta-arrestin recruitment, or spatial signaling predict resistance to defined lipotoxic insults rather than merely differences in pharmacokinetic persistence.

A fourth priority is development of human mechanistic endpoints capable of linking changes in lipid handling to organ function. MRI-PDFF can quantify hepatic lipid burden, whereas circulating lipidomics and cholesterol efflux capacity may provide complementary systemic measures of lipid remodeling [57,58,59,60]. Dynamic meal- or glucose-based assessment of beta-cell function can be integrated with these measurements to test whether changes in lipid-related endpoints mediate improvements in insulin secretion independently of weight loss. Cell-specific approaches, including beta-cell-derived extracellular vesicles, remain exploratory and require analytical and biological validation before they can be considered response biomarkers [61].

Combination and multi-target strategies intended to complement GLP-1R-mediated metabolic effects should currently be regarded as hypothesis-generating rather than established approaches for alleviating lipotoxicity. In preclinical models, combined exenatide and olmesartan treatment has been evaluated for renal injury and albuminuria in obese insulin-resistant rats [62]. Similarly, liraglutide has been studied in combination with OLHHA, a dual PPARα agonist and peripheral CB1 antagonist, in experimental metabolic liver disease [63]. A distinct proof-of-concept approach has integrated a GLP-1 agonist peptide with an anti-PCSK9 antibody in a single fusion molecule, demonstrating combined GLP-1R agonist and LDL-lowering pharmacology in preclinical models [64]. However, these studies do not establish additive anti-lipotoxic efficacy in humans. Future studies should therefore evaluate selected combination or multi-target strategies prospectively using mechanism-specific endpoints and appropriate comparator groups rather than assuming additive benefit from their individual metabolic effects.

## 13. Conclusions

GLP-1 receptor agonist therapy has clinically established systemic effects that can reduce major drivers of lipotoxic stress, including reduced energy intake and body weight, improved glycemic control, and beneficial cardiovascular, hepatic, and kidney outcomes in selected clinical settings [5,6,43,55,56]. These systemic effects provide the strongest translational basis for reducing lipid exposure to metabolically vulnerable tissues. Beyond these established clinical effects, experimental studies with individual GLP-1 receptor agonists, particularly exendin-4 and liraglutide, support tissue-specific cytoprotective mechanisms in pancreatic beta cells, including attenuation of ER stress, oxidative injury, defective autophagy, mitochondrial dysfunction, and apoptosis [16,17,18,19,20,21,22,23,24,25,26,27,28,29,30]. Recent mechanistic work further implicates spatially organized GLP-1R signaling at ER–mitochondria contact sites [31].

More specific lipid-handling mechanisms remain promising but incompletely validated. In preclinical beta-cell models, exendin-4 induces ABCA1 through CaMKK/CaMKIV/PREB-dependent and related pathways [34,35,37]; however, this mechanism has not been established as a class-wide property of clinically used GLP-1RAs, and direct validation of GLP-1RA-induced ABCA1-dependent cholesterol efflux in primary human beta cells remains lacking. In the liver, preclinical cell-resolved and genetic loss-of-function evidence in mouse MASH models identifies pericentral LSEC GLP-1R signaling as an important contributor to weight-loss-independent semaglutide-mediated improvements in steatosis, fibrosis and immune remodeling [44]. These findings support a receptor-dependent intrahepatic mechanism in mice, but corresponding GLP-1R distribution, intercellular signaling and causal relevance in human MASH remain to be demonstrated. Direct GLP-1R-dependent actions in human hepatocytes also remain uncertain [49,50]. Emerging mechanisms involving renal lipid handling are similarly supported predominantly by preclinical evidence and should not be inferred from demonstrated clinical kidney protection alone.

Taken together, the available evidence supports an evidence-calibrated framework in which established systemic metabolic effects are complemented by experimentally supported tissue-specific mechanisms and by emerging mechanistic hypotheses that require further validation in humans. Defining which pathways are shared across clinically used agonists, which require cell-autonomous GLP-1R signaling, and which independently mediate organ protection beyond weight loss and glycemic improvement will be essential for translating these mechanistic observations into clinically meaningful biomarkers and therapeutic strategies.

## Figures and Tables

**Table 1:** Mechanistic and translational evidence for GLP-1 receptor agonist-associated relief of lipotoxic stress.

Target/Process	Specific Agonist/Ligand	Principal Mechanistic Response	Experimental Evidence *	Receptor Dependence/Causality	Translational Interpretation
**Beta-cell ER stress and apoptosis**	Exendin-4; liraglutide	Reduced ER-stress burden and apoptosis; BiP/JunB induction; modulation of PERK/eIF2α and IRE1 signaling	**CL; PH; A**	GLP-1R-associated; detailed downstream mechanisms predominantly established in experimental models	Preclinical evidence is substantial; exendin-4 protection has also been demonstrated in human islets, but clinical mediation of beta-cell preservation by these pathways is unproven
**Beta-cell autophagy**	Liraglutide	Increased autophagic turnover and protection against palmitate-induced injury; AMPK-associated autophagic signaling	**CL; A**	Mechanistic evidence predominantly pharmacological; receptor-specific causal validation remains limited	Predominantly preclinical; no direct human-islet or clinical validation of autophagy as a mediator of GLP-1RA benefit
**Oxidative and mitochondrial stress**	Predominantly exendin-4	Nrf2 activation; reduced JNK/GSK3β signaling; preservation of mitochondrial coupling; PPARδ/UCP2-associated responses	**CL; A**	Pathway-specific perturbation supports causality in selected models	Preclinical and compound-specific; human beta-cell relevance remains to be established
**Fatty-acid stress signaling**	Exendin-4	Attenuation of GPR40–MKK4/7–JNK and SREBP-1c/C/EBPβ-associated stress responses	**CL; A**	Pathway inhibition/perturbation supports mechanistic involvement in experimental models	Preclinical; should not be generalized to all clinically used GLP-1RAs
**Endosomal GLP-1R–ERMCS signaling**	Predominantly exendin-4; additional agonists assessed for receptor trafficking	Endosomal GLP-1R–VAPB–SPHKAP assembly; localized cAMP/PKA signaling; MICOS-dependent mitochondrial remodeling	**CL; PH; A**	Strong receptor- and pathway-resolved evidence in experimental systems	Mechanistically advanced preclinical evidence, including primary human islets; protection from defined lipotoxic stress and comparative effects among clinical agonists remain incompletely established
**Beta-cell cholesterol efflux/ABCA1**	Exendin-4; native GLP-1 in selected studies	Increased ABCA1 expression and cholesterol efflux via CaMKK/CaMKIV/PREB, AMPK/PARP-1/LXR, and miRNA-associated pathways	**CL; A; HM †**	Specific CaMKK/CaMKIV/PREB sequence supported primarily by the authors’ group; independent studies support convergence on ABCA1 through other upstream pathways	ABCA1 biology is supported by human genetic data, but GLP-1RA-induced ABCA1 expression/efflux has not been directly validated in primary human beta cells
**Hepatocyte-model lipid handling**	Exendin-4; native GLP-1 in selected studies	ABCA1 induction; reduced lipogenesis; increased autophagy and fatty-acid oxidation	**CL; A**	Pharmacological evidence supports GLP-1R-associated signaling in some model systems, but physiologically relevant human hepatocyte GLP-1R expression remains uncertain	Model-dependent preclinical evidence; should not be interpreted as an established direct human-hepatocyte GLP-1R mechanism
**LSEC-centered hepatic signaling**	Semaglutide	Endothelial GLP-1R-dependent signaling with downstream effects on steatosis, fibrosis, and immune remodeling	**A**	**Strong genetic causal evidence in mouse MASH models** through endothelial Glp1r loss-of-function approaches	Receptor-resolved preclinical evidence; corresponding GLP-1R distribution and causal signaling in human LSECs remain unproven
**Clinical hepatic protection/systemic unloading**	Semaglutide; broader GLP-1RA evidence	Reduced substrate delivery and liver fat; improvement in steatohepatitis and fibrosis-related histological endpoints	**HM; RCT**	Clinical benefit established; contribution of direct intrahepatic receptor signaling cannot be inferred from RCTs	Human clinical efficacy established for selected agents/settings; cellular mechanism remains incompletely resolved
**Renal lipid stress**	Exendin-4; liraglutide; semaglutide for clinical kidney outcomes	Increased ABCA1-associated cholesterol efflux; altered fatty-acid metabolism; reduced ectopic lipid accumulation and ferroptotic stress	**CL; A; RCT ‡**	Direct renal lipid-handling mechanisms predominantly preclinical; receptor dependence varies by model	Clinical kidney protection is established for semaglutide in T2D/CKD, but mediation through renal lipotoxicity has not been demonstrated

**Abbreviations:** ABCA1, ATP-binding cassette transporter A1; AMPK, AMP-activated protein kinase; BiP, binding immunoglobulin protein; cAMP, cyclic adenosine monophosphate; C/EBPβ, CCAAT/enhancer-binding protein β; CaMKK, Ca2+/calmodulin-dependent protein kinase kinase; CaMKIV, Ca2+/calmodulin-dependent protein kinase IV; CKD, chronic kidney disease; eIF2α, eukaryotic initiation factor 2α; ER, endoplasmic reticulum; ERMCS, endoplasmic reticulum–mitochondria contact site; FFAR1/GPR40, free fatty acid receptor 1/G protein-coupled receptor 40; GLP-1R, GLP-1 receptor; GLP-1RA, GLP-1 receptor agonist; GSK3β, glycogen synthase kinase 3 beta; IRE1, inositol-requiring enzyme 1; JunB, JunB proto-oncogene, AP-1 transcription factor subunit; JNK, c-Jun N-terminal kinase; LSEC, liver sinusoidal endothelial cell; LXR, liver X receptor; MASH, metabolic dysfunction-associated steatohepatitis; MICOS, mitochondrial contact site and cristae organizing system; miRNA, microRNA; MKK4/7, mitogen-activated protein kinase kinases 4/7; Nrf2, nuclear factor erythroid 2-related factor 2; PARP-1, poly(ADP-ribose) polymerase 1; PERK, protein kinase R-like ER kinase; PKA, protein kinase A; PPARδ, peroxisome proliferator-activated receptor delta; PREB, prolactin regulatory element-binding protein; SPHKAP, sphingosine kinase type 1-interacting protein; SREBP-1c, sterol regulatory element-binding protein 1c; T2D, type 2 diabetes; UCP2, uncoupling protein 2; VAPB, vesicle-associated membrane protein-associated protein B. * Evidence domains: CL, cell-line evidence; PH, primary human-cell or human-islet evidence; A, animal evidence; HM, human mechanistic, genetic, or observational evidence; RCT, randomized clinical evidence. Evidence domains indicate the type of supporting evidence and should not be interpreted as a simple ordinal hierarchy. In particular, clinical outcome evidence does not by itself establish the cellular mechanism responsible for that outcome. † Human genetic data support the importance of ABCA1 for beta-cell function but do not establish GLP-1RA-induced ABCA1 regulation in human beta cells. ‡ Randomized clinical evidence supports kidney outcome benefits of semaglutide but does not establish modulation of renal lipotoxicity as the causal mechanism.

## Data Availability

No new data were created or analyzed in this study. Data sharing is not applicable to this article.

## Supplementary Materials

The following supporting information can be downloaded at: https://www.mdpi.com/article/10.3390/ijms27188378/s1.
