# Supplementary material for: GLP-1 Receptor Agonists as Molecular Relievers of Lipotoxic Stress: From Pancreatic Beta-Cell Cholesterol Efflux to Systemic and Tissue-Specific Metabolic Protection
Source: Int J Mol Sci. 2026 Sep 20;27(18):8378. doi: 10.3390/ijms27188378 (PMC13607562; doi:10.3390/ijms27188378)
Supplement: Supplementary file 1 [file ijms-27-08378-s001.zip › ijms-4560981-supplementary.pdf]

Supplementary Table S1. Principal GLP-1R Ligands and Agonists Discussed in This Review and Their U.S. Regulatory Context

Ligand/agonist	Role in this review	U.S. FDA regulatory context
**Exendin-4 / exenatide**	Major experimental ligand in β-cell ER stress, oxidative stress, ABCA1, ERMCS and hepatic studies	Exenatide is the synthetic pharmaceutical form of exendin-4 and is FDA-approved for T2D; initial U.S. approval 2005. Studies experimentally using exendin-4 are identified as such and are not generalized to the entire GLP-1RA class.
**Liraglutide**	β-cell ER stress/autophagy; hepatic lipid handling; renal mechanisms	FDA-approved GLP-1RA; initial U.S. approval 2010.
**Semaglutide**	Clinical MASH evidence; mouse LSEC genetic studies; systemic clinical outcomes	FDA-approved GLP-1RA; initial U.S. approval 2017; multiple approved formulations/indications.
**Native GLP-1**	Endogenous ligand; ABCA1 and receptor-trafficking mechanistic studies	Endogenous ligand; not an FDA-approved therapeutic GLP-1RA.
**Exendin-F1 / exendin-D3**	Experimental biased agonists used to study GLP-1R internalization and spatial signaling	Experimental research ligands; not FDA-approved therapeutic products.
**N-terminally modified GLP-1R agonists (e.g., Ac-GLP-1, GLP1-Y11, Ac-semaglutide)**	Structural/biased-signaling studies	Experimental modified ligands; not FDA-approved therapeutic products. FDA approval of parent semaglutide does not extend to these modified experimental molecules.

Abbreviations: ABCA1, ATP-binding cassette transporter A1; ER, endoplasmic reticulum; ERMCS,

endoplasmic reticulum–mitochondria contact site; FDA, U.S. Food and Drug Administration; GLP-1R, glucagon-like peptide-1 receptor; GLP-1RA, glucagon-like peptide-1 receptor agonist; LSEC, liver sinusoidal endothelial cell; MASH, metabolic dysfunction-associated steatohepatitis; T2D, type 2 diabetes.

U.S. FDA regulatory status and initial approval dates were verified using Drugs@FDA and FDA approval documents (accessed September 2026): exenatide/Byetta (NDA 021773; April 28, 2005), liraglutide/Victoza (NDA 022341; January 25, 2010), and semaglutide/Ozempic (NDA 209637; December 5, 2017). Regulatory approval and current marketing availability are distinct; some historically approved GLP-1RA products or formulations have subsequently been discontinued or are no longer marketed in the United States.
